# Relationship between health-related quality of life, comorbidities and acute health care utilisation, in adults with chronic conditions

**DOI:** 10.1186/s12955-015-0260-2

**Published:** 2015-05-29

**Authors:** Anastasia F. Hutchinson, Marnie Graco, Tshepo Mokuedi Rasekaba, Sumit Parikh, David John Berlowitz, Wen Kwang Lim

**Affiliations:** Northern Clinical Research Centre, Northern Health, 185 Cooper Street, 3076 Epping, Victoria Australia; Centre for Quality Patient Safety Research, School of Nursing & Midwifery, Deakin University, Victoria, Australia; Institute for Breathing and Sleep, Austin Health, Melbourne, Australia; Primary Care Research Unit, General Practice and Primary Health Care Academic Unit, The University of Melbourne, Melbourne, Australia; Department of Medicine and Aged Care, Northern Health & Department of Medicine, The University of Melbourne, Melbourne, Australia

**Keywords:** Health-related quality of life, Acute healthcare utilisation, Chronic disease management, Ambulatory care sensitive conditions, Chronic obstructive pulmonary disease, Chronic heart failure, Diabetes, Aged care

## Abstract

**Background:**

There is increased interest in developing multidisciplinary ambulatory care models of service delivery to manage patients with complex chronic diseases. These programs are expensive and given limited resources it is important that care is targeted effectively. One potential screening strategy is to identify individuals who report the greatest decrement in health related quality of life (HRQoL) and thus greater need. The aim of this study was to explore the relationship between HRQoL, comorbid conditions and acute health care utilisation.

**Methods:**

A prospective, longitudinal cohort design was used to evaluate the impact of HRQoL on acute care utilisation rates over three-years of follow-up. Participants were enrolled in chronic disease management programs run by a metropolitan health service in Australia. Baseline data was collected from 2007–2009 and follow-up data until 2012. Administrative data was used to classify patients’ primary reasons for enrolment, number of comorbidities (Charlson Score) and presentations to acute care. At enrolment, HRQoL was measured using the Assessment of Quality of Life (AQoL) instrument, for analysis AQoL scores were dichotomised at two standard deviations below the population norm.

**Results:**

There were 1999 participants (54 % male) with a mean age of 63 years (range 18–101), enrolled in the study. Participants’ primary health conditions at enrolment were: diabetes 915 (46 %), chronic respiratory disease 463 (23 %), cardiac disease 260 (13 %), peripheral vascular disease, and 181 (9 %) and aged care 180 (9 %). At 1-year multivariate logistic regression models demonstrated that AQOL utility score was not predictive of acute care presentations after adjusting for comorbidities. Over 3-years an AQoL utility score in the lowest quartile was predictive of both ED presentation (OR 1.58, 95 % CI, 1.16–2.13, p = 0.003) and admissions (OR 1.67, 95 % CI.1.21 to 2.30, p = 0.002) after adjusting for differences in age and comorbidities.

**Conclusion:**

This study found that both HRQoL and comorbidities were predictive of subsequent acute care attendance over 3-years of follow-up. At 1-year, comorbidities was a better predictor of acute care representation than HRQoL. To maximise benefits, programs should initially focus on medical disease management, but subsequently switch to strategies that enhance health independence and raise HRQoL.

## Background

In response to the worldwide growth in the prevalence of chronic disease, there is increased interest in developing ambulatory models of care that will improve patient outcomes and minimise demand on emergency departments and acute inpatient services [1–3]. Over the past 15 years Australian health authorities have funded a wide range of programs to improve the management of patients with chronic disease that requires acute care [4]. These initiatives typically identify patients following acute care attendance for conditions such as chronic heart failure (CHF), chronic obstructive lung disease (COPD), or diabetes and offer interventions such as: exercise rehabilitation, self-management education and support, community-outreach and case-management services. To ensure effective use of limited resources it is important that these programs enrol those patients who will benefit the most and/who are at high risk of re-attendance for acute care management. One of the challenges clinicians therefore face is identifying these high-risk individuals, so that the programs can be targeted effectively to those patients with the highest level of need [5].

Internationally there have been a range of attempts at developing prediction algorithms that identify patients at high risk of early acute care readmission who would benefit from increased ambulatory care services post discharge [5, 6]. For example, a US study found that age, sex, ethnicity, number of previous admissions, and clinical condition were indicators of readmission risk and that those scoring ≥80 (on a 0–100 scale) had an 84 % likelihood of being readmitted over the following 12-months [7]. Although numerous risk stratification tools have been developed and validated for individual disease groups [8–11] there is a lack of generic measures that can be used across different conditions, ages and complexity of disease to predict individuals at high risk of readmission to acute care. Many risk stratification instruments focus on measures of disease severity and fail to measure the patient factors that modify subsequent health care utilisation behaviour [12–14]. Furthermore, identification of individuals with advanced disease who may benefit from advance care planning or more palliative approaches to disease management is reportedly poor [15]. To address these questions this study was undertaken to evaluate whether a generic measure of health related quality of life (HRQoL) was predictive of subsequent acute health care utilisation in a heterogeneous patient group [16].

The Northern Alliance Hospital Admission Program (NA-HARP) service provides care to a socio-economically disadvantaged population living in the northern metropolitan region of Melbourne, Australia [16, 17]. The service offers multidisciplinary disease management and short-term care coordination to individuals with chronic disease (ischaemic heart disease (IHD), CHF, COPD, asthma, diabetes, peripheral vascular disease (PVD) and chronic wounds) and older adults with complex aged care related needs [4]. The aim of this analysis was to describe the HRQoL of adults enrolled in the NA-HARP programs and to explore the relationship between HRQoL, comorbid conditions and acute health care utilisation over the three years following enrolment.

## Methods

### Setting

The NA-HARP program provides multidisciplinary case-management, exercise rehabilitation, disease self-management education and support, ambulatory care management of complex wounds and care coordination for patients with complex psychosocial needs. The multidisciplinary team includes: specialist physicians, physiotherapy, health psychology, occupational therapy, social workers and specialist nurse consultants. The program includes separate streams of care for patients with (1) chronic respiratory diseases (asthma and COPD), (2) cardiac conditions (IHD and CHF), (3) diabetes, (4) peripheral vascular disease and complex wounds, and (5) complex psychosocial and aged care needs.

Eligibility for the program was patients with one or more chronic conditions that placed them at high risk of emergency department attendance or acute care admission [2–4]. Although referral to the program was accepted from both primary and acute health care providers, priority was given to patients who had been recently discharged from acute care. At enrolment clinicians within each stream conducted a comprehensive needs assessment and developed a care-plan which included a suite of interventions tailored to the individuals’ needs.

### Study design

A prospective, longitudinal cohort design was used to evaluate whether a measure of HRQoL obtained at enrolment was predictive of acute care re-attendance over a three year follow-up period. Baseline data was collected from September 2007–2009 and follow-up data was obtained until December 2012. This project was approved by the Northern Health institutional human research ethics committee, the requirement for written informed consent was waived.

### Participants

Participants were consecutive patients enrolled in the NA-HARP service who completed the Assessment of Quality of Life (AQoL) at program enrolment.

### Data collection

From 2007–2009, NA-HARP clinicians distributed AQoL surveys by mail or directly to participants following their first assessment visit. Surveys were returned to the service by reply paid post. Although the AQoL has been translated into several languages, professional interpreters were also made available to assist participants who spoke a language other than English and for those with limited literacy.

### Outcomes

The AQoL is a validated, multi-attribute utility instrument designed to assess HRQoL that is sensitive to a range of patient conditions and care models [18]. It measures five dimensions: “Illness”, “independent living”, “social relationships”, “physical senses”, and “psychological wellbeing”. These scales are scored as proportions on a 0.00–1.00 scale. Scores from the last four dimensions are combined using a multiplicative model weighted with community values to compute the utility index, which is suitable for use in cost-utility analysis [18]. The utility scores range from −0.04 (HRQoL worse than death), 0.00 (representing death equivalent states), to 1.00 (full HRQoL), [19]. The AQOL is designed to be self-administered taking an average of five to seven minutes to complete. Population norms are available, which allow the results to be interpreted relative to an age-matched Australian population average [20, 21]. The published minimum important difference (MID) is 0.06 utilities [20].

Administrative data was used to classify patients’ primary reasons for enrolment into the NA-HARP service, and the Charlson Comorbidity score (Charlson) at baseline was calculated based on patients’ primary and secondary ICD-10 diagnoses codes from acute care admissions prior to the patient’s enrolment in the service [22]. Charlson weights were allocated to ICD-10 scores using the algorithm developed by Quan e al. [23, 24]. Comorbidities were summarised as a Charlson Score based on the original scoring weights published by Charlson [24] the total Charlson score ranges between o and a maximum of 24 points), the age-adjusted Charlson Index was calculated adding one additional point for each decade greater than 40 to 49 years.

At the end of the follow-up period, the number of readmissions to acute care over the three years following enrolment were obtained from the regional health service’s administrative dataset and verified by audit of individual patient medical records. Data was obtained on both the number, duration and time (measured in years) to these outcomes and acute care utilisation was measured as: emergency department (ED) readmission rate, inpatient readmission rate, acute care length of stay and total inpatient bed-days per annum. When calculating acute health care utilisation rates over the three year follow-up, the denominator was adjusted for deaths or patients who were lost to follow-up.

### Data analysis

Administrative and AQoL data were retrieved for patients enrolled in the service between September 2007 and September 2009. Continuous data were summarised as means and standard deviations (SDs); categorical data as frequencies and percentage, differences in proportions were analysed with Chi-square (χ^2^) tests, differences in continuous outcomes using T-tests. Differences in age and Charlson score across the NA_HARP program streams was analysed using ANOVA.

Mean AQoL scores were compared with published population data across age deciles using Welch’s approximate t-tests to control for differences in data distributions [24]. As Bartlett’s test for equal variances between groups (according to primary health condition) showed statistically significant variation, and the AQoL utilities were not normally distributed; the Kruskal-Wallis non-parametric AVOVA was used to analyse differences in baseline AQoL utilities and AQoL dimension scores according age group, primary condition, service type and comorbidity scores. In the absence of any known cut points, the AQoL dimensions were entered as continuous variables into the model. Statistical significance was set at p < 0.05.

Univariate logistic regression was used to assess which factors were predictive of acute health care utilisation over a three year follow-up period [25]. AQoL scores were dichotomised at two standard deviations below the population norm (ie: (<0.37/≥0.37) [20]. and the Charlson Score was summarised as a four level ordinal variable (0 or 1, 2 or 3, 4 or 5 and 6 or more). Multivariate logistic regression, using a forward stepwise model, was used to assess whether lower AQoL scores at enrolment were predictive of patients who required either an emergency department attendance or an inpatient readmission within 12-months and three years of enrolment after adjusting for age, and Charlson Score. As there were significant differences in AQoL scores according to Charlson score, an interaction term was added into the model to test the interaction between these factors. Marginal analysis was used to test the change in probability of acute care utilisation for AQoL utility values (<0.37 versus ≥0.37) for each level of the Charlson Score in the models [26]. The Hosmer and Lemeshow statistic was used to test the goodness of fit of the models [27] and the models’ predictive accuracy were summarised using sensitivity, specificity and positive and negative predictive values.

Data were analysed using STATA version 12, Statacorp Texas USA [28].

## Results

A consecutive sample of 1999 individuals enrolled in the NA-HARP program between 2007 and 2009 had the AQoL measured at enrolment into the program and were included in this study. The mean follow-up time for study participants was 2.9 years (SD 0.55) and 180 (9 %) died during follow-up. The mean age of participants was 63 years (range 18–101) and 54 % were male. Sixty-six percent were born in a country other than Australia, 574 (29 %) spoke a language other than English, and 325 (16 %) required an interpreter for health care appointments (Table 1). Over half of the participants (1310, 66 %) had an age adjusted Charlson Index of ≥ 3 and 448 (22 %) had a Charlson Index of ≥ 7, indicating a substantial comorbidity burden.

**Table 1:** Participant characteristics

Participant characteristics		*N* = 1999
Gender	Male	1071 (54 %)
	Female	928 (46 %)
Age	Mean (SD) years	63 (14.4)
	Range	18–101
Other household members	Lived alone	366 (18 %)
	Lived with family	1495 (75 %)
	Lived with others	93 (5 %)
	Unknown	45 (2 %)
Accommodation Type	Private residence	1857 (93 %)
	Supported accommodation	63 (3 %)
	Residential aged care facility	22 (1 %)
	Other	57 (3 %)
Primary health condition	Diabetes	928 (46 %)
	Chronic respiratory	463 (23 %)
	Cardiac	277 (14 %)
	Peripheral vascular disease/Diabetic Foot/Wounds	191 (10 %)
	Neurological/Musculoskeletal/Functional status	140 (7 %)
Country of Birth	Australia/New Zealand	877 (44 %)
	United Kingdom/Ireland	77 (4 %)
	Europe	724 (36 %)
	Middle East	140 (7 %)
	Other	173 (9 %)
Charlson Comorbidity Score	0/1	956 (48 %)
	2/3	494 (25 %)
	4/5	288 (14 %)
	≥6	261 (13 %)
Charlson Index	0	99 (5 %)
	1/2	589 (29.5 %)
	3 /4	531 (26.7 %)
	5/6	331 (16.6 %)
	≥7	448 (22.4 %)
Service Type	Diabetes	915 (46 %)
	PVD/ Diabetic Foot/Wound	181 (9 %)
	Respiratory	463 (23 %)
	Asthma (215)	
	COPD (188)	
	Other Respiratory (60)	
	Cardiac	260 (13 %)
	Ischaemic Heart Disease (136)	
	Chronic Heart Failure (122)	
	Aged care/complex psychosocial	180 (9 %)
Acute health care utilisation	ED presentations - Mean (SD)	0.77 (1.87)
1 year following enrolment/patient	Emergency inpatient admissions - Mean (SD)	0.50 (1.41)
	Elective inpatient admissions - Mean (SD)	0.30 (0.99)
	Total Bed-days - Mean (SD)	2.50 (8.10)
Over 3 years following enrolment/patient	ED presentations - Mean (SD)	1.96 (3.62)
	Emergency inpatient admissions - Mean (SD)	1.29 (2.70)
	Total Bed-days - Mean (SD)	6.73 (16.6)

Participants were enrolled from the following streams within the NA-HARP program: diabetes 915 (46 %), chronic respiratory disease 463 (23 %), cardiac disease 260 (13 %), peripheral vascular disease, and wound management services 181 (9 %) and aged care/complex case management services 180 (9 %), (Table 1). Analysis of covariance demonstrated that there were significant differences in age (F = 70.5, df = 4, p < 0.001) and Charlson Score (F = 17.5, df = 4, p < 0.001) across the program streams. The respiratory, cardiac and diabetes streams overall included younger adults with 142 patients aged 18–39 years, in contrast to five individuals in this age group in the PVD/wound stream and none in the aged care/complex case-management stream. The mean Charlson scores across steams of care were: respiratory 2.5 (SD 2.5), cardiac 2.4 (SD .7) and diabetes 2.3 (SD 2.3), in comparison to the complex case management 3.2 (SD 3.0) and PVD 3.8 (SD 3.2) streams.

The mean HRQoL for all participants was AQoL 0.55 (SD 0.32, range −0.04 to 1). A HRQoL level considered “worse than death” (AQoL < 0) was reported by 56 (3 %) of participants. When compared with age-matched population norms, study participants reported significantly worse HRQoL. These differences exceeded the published minimum important difference (MID) of 0.06 for the AQoL for those aged 30 years and older (Table 2).

**Table 2:** Participants’ HRQoL (AQoL utility) compared with age-adjusted population values

Age group	Participants			Population^a^			Statistics^b^
	*N*	Mean	SD	*N*	Mean	SD	
18–29 years^c^	38	0.84	0.20	1325	0.86	0.19	df = 37, p = 0.50
30–39 years	108	0.72	0.26	1681	0.84	0.21	df = 107, p < 0.001
40–49 years	211	0.61	0.30	1382	0.81	0.23	df = 210, p < 0.001
50–59 years	376	0.59	0.31	1295	0.80	0.24	df = 375, p < 0.001
60–69 years	543	0.59	0.30	1245	0.80	0.22	df = 542, p < 0.001
70–79 years	545	0.47	0.30	912	0.76	0.23	df = 544, p < 0.001
80+ years^d^	174	0.31	0.27	357	0.70	0.26	df = 173, p < 0.001

Notes:

^a^ = Source: Hawthorne, Korn & Richardson (2013)

^b^ = Independent t-test

^c^ = Includes 6 cases <20 years

^d^ = The population was restricted to those 80 to 85 years

Table 3 shows the HRQoL of participants by primary health condition and Charlson comorbidity score. There were statistically significant differences between the different primary health conditions across the dimensions of the AQoL and in overall AQoL utility scores. Participants with asthma, ischaemic heart disease or diabetes (without peripheral vascular complications) had higher overall HRQoL (AQoL utilities 0.57–0.62) than those with CHF, COPD, peripheral vascular disease with complications and complex wounds (AQoL utilities 0.40-0.45) or those with aged care and complex needs (AQoL utility 0.33). Participants with PVD reported the lowest scores in the illness dimension of the AQoL (0.19), compared to scores of 0.46 and 0.49 for asthma and IHD respectively. Those in the aged care with complex needs group reported the lowest scores on the independent living dimension of the AQoL (0.49) compared to scores of 0.82 to 0.84 amongst those with asthma, IHD and diabetes. Differences between medical condition groups on the social relationships, physical senses and psychological wellbeing dimensions were smaller, although the differences between the lowest and highest scoring groups did reach the MID.

**Table 3:** Differences in HRQoL (AQoL) dimensions across primary condition and Charlson comorbidity scores

			Mean (SD) AQoL dimension scores^a^					AQoL
		*N*	Ill	IL	SR	PS	PW	utility
Primary health condition	Asthma	215	0.46 (0.30)	0.82 (0.26)	0.85 (0.22)	0.93 (0.11)	0.83 (0.17)	0.61 (0.30)
	COPD/other	248	0.39 (0.27)	0.68 (0.30)	0.79 (0.23)	0.88 (0.12)	0.80 (0.19)	0.45 (0.27)
	Chronic Heart Failure	122	0.28 (0.24)	0.68 (0.32)	0.79 (0.24)	0.87 (0.15)	0.78 (0.20)	0.45 (0.31)
	Ischaemic Heart Disease	136	0.49 (0.23)	0.82 (0.26)	0.84 (0.20)	0.91 (0.10)	0.82 (0.15)	0.57 (0.27)
	Diabetes	915	0.34 (0.25)	0.84 (0.26)	0.88 (0.21)	0.93 (0.10)	0.82 (0.20)	0.62 (0.31)
	Peripheral Vascular Disease	151	0.19 (0.22)	0.63 (0.33)	0.76 (0.27)	0.86 (0.15)	0.76 (0.22)	0.40 (0.32)
	Wound care	30	0.43 (0.29)	0.60 (0.37)	0.78 (0.22)	0.88 (0.15)	0.83 (0.16)	0.43 (0.33)
	Aged Care	180	0.30 (0.25)	0.49 (0.33)	0.72 (0.29)	0.86 (0.14)	0.79 (0.19)	0.33 (0.27)
	Kruskal-Wallis ANOVA Test		χ^2^ = 141.6 *p* < 0.001	χ^2^ = 252.1 *p* < 0.001	χ^2^ = 132.2 *p* < 0.001	χ^2^ = 102.4 *p* < 0.001	χ^2^ = 29.52 *p* < 0.001	χ^2^ = 208.9 *p* < 0.001
Statistics	0/1	955	0.43 (0.27)	0.84 (0.25)	0.86 (0.21)	0.92 (0.11)	0.83 ( 0.18)	0.61 (0.30)
Charlson Score	2/3	494	0.32 (0.24)	0.76 (0.29)	0.84 (0.23)	0.91 (0.11)	0.81 (0.20)	0.54 (0.31)
	4/5	288	0.28 (0.23)	0.69 (0.31)	0.80 (0.25)	0.89 (0.12)	0.80 (0.19)	0.48 (0.31)
	≥6	262	0.23 (0.24)	0.59 (0.35)	0.77 (0.27)	0.87 (0.15)	0.77 (0.21)	0.39 (0.31)
	Kruskal-Wallis ANOVA Test		χ^2^ = 182.8 *p* < 0.001	χ^2^ = 153.4 *p* < 0.001	χ^2^ = 45.5 *p* < 0.001	χ^2^ = 40.3 *p* < 0.001	χ^2^ = 24.3 *p* < 0.001	χ^2^ = 118.9 *p* < 0.001

^a^ = Ill: Illness; IL: Independent living; SR: Social relationships; PS: Physical senses; PW: Psychological wellbeing; SD = Standard Deviation

Across all dimensions of the AQoL, HRQoL decreased as the number of comorbidities increased, the differences between those scoring 0–1 on the Charlson and those with six or more comorbidities on the Charlson was 0.22 (p < 0.001) for overall HRQoL. Differences in AQoL dimensions between those with the lowest (0–1) and highest (≥6) Charlson scores reached the MID for: the illness (difference 0.23, p < 0.001), independent living (difference 0.25, p < 0.001), psychological well-being (difference 0.22, p < 0.001), and social relationships (difference 0.09, p < 0.001) dimensions.

### Acute health care utilisation

Thirty-two percent (640) of participants presented to ED during the first 12-months following enrolment and 1081 (54 %) presented at least once over three years. The number of ED presentations per patient in the first year ranged from 0–31 (mean 0.96 (SD 1.64)) and over three years ranged from 0–52 per patient per year (mean 0.83 SD 2.17), (Table 1). Inpatient admission was required for 489 (25 %) of participants during the first 12-months following enrolment and for 841 (42 %) over three years. The number of readmissions per patient ranged from 0 through to 20 (mean 0.50 (SD 1.41)) in the first year and 0 to 33 per patient per year over three years (mean 1.29 (SD 2.69)).

Additionally, each two point increase in the Charlson score was associated with at 70 % increase in the odds of ED attendance in the first 12 months post enrolment (OR 1.70 (95 % CI: 1.56 to 1.86, p < 0.001) and almost twice the odds of emergency inpatient admission (OR 1.98, 95 % CI: 1.80 to 2.18, p < 0.001). These differences were sustained over the three years of follow-up with each 2 point increase in the Charlson Score being associated with a 40 % increase in the odds of ED attendance (OR 1.40, 95 % CI: 1.30 to 1.51, P < 0.001), a 70 % increase in the odds of non-elective inpatient admission (OR 1.70, 95 % CI, 1.57 to 1.84, p < 0.001) and 64 % increase in average annual beddays over three years (OR 1.64, 95 % CI 1.52 to 1.77, p < 0.001).

An AQoL utility score < 0.37 was associated with an approximately two fold increase in emergency presentation (OR 1.96, 95 % CI, 1.61 to 2.3, p < 0.001) or inpatient admission (OR 2.20, 95 % CI, 1.78 to 2.72, p < 0.001) over one and three years (OR 2.05, 95 % CI: 1.69 to 2.50, p < 0.001 and OR 2.36 95 % CI, 1.95 to 2.86, p < 0.001), (Table 4).

**Table 4:** Univariate logistic regression models: predicting acute health care utilisation

Predictor	OR	95 % CI	*P* Value
ED presentation with 1 year			
AQOL utility score <0.37	1.96	1.61 to 2.3	<0.001
Charlson Score	1.70	1.56 to 1.86	<0.001
Age group	1.27	1.13 to 1.44	<0.001
Inpatient admission within 1 year			
AQOL utility score <0.37	2.20	1.78 to 2.72	<0.001
Charlson Score	1.98	1.80 to 2.18	<0.001
Age group	1.52	1.33 to 1.73	<0.001
ED presentations within 3 years			
AQOL utility score <0.37	2.05	1.69 to 2.50	<0.001
Charlson Score	1.72	1.57 to 1.88	<0.001
Age group	1.26	1.12 to 1.41	<0.001
Inpatient admission within 3 years			
AQOL utility score <0.37	2.36	1.95 to 2.86	<0.001
Charlson Score	2.06	1.88 to 2.27	<0.001
Age group	1.64	1.46 to 1.85	<0.001

Age group: ordinal variable: 18–50, 51–70, 71–84, and ≥85 years

Multivariate logistic regression models were constructed to determine whether HRQoL measured by the AQoL predicted acute health care utilisation over one and three years of follow-up (Table 5). To adjust for differences across the NA_HARP program streams of care, age, and Charlson score were included in the models. Age was not predictive of acute health care utilisation in either the one or three year multivariate models.

**Table 5:** Multivariate logistic regression models: predicting acute health care utilisation

ED presentation with 1 year				ED presentations within 3 years			
Predictor	OR	95 % CI	*P* Value	Predictor	OR	95 % CI	*P* Value
AQOL utility score <0.37	1.35	0.95 to 1.91	0.094	AQOL utility score <0.37	1.58	1.16 to 2.13	0.003
Charlson Score (2–3)	1.48	1.09 to 2.00	0.011	Charlson Score (2–3)	1.71	1.32 to 2.23	<0.001
Charlson Score (4–5)	2.39	1.66 to 3.45	<0.001	Charlson Score (4–5)	1.96	1.39 to 2.76	<0.001
Charlson Score (≥6)	4.91	3.26 to 7.40	<0.001	Charlson Score (≥6)	6.36	3.96 to 10.20	<0.001
Correct classification: 70.0 %; Hosmer and Lemeshow χ^2^ (9) = 11.53, *p* = 0.117.				Correct classification: 62.09 %; Hosmer and Lemeshow χ^2^ (8) = 34.00, *p* < 0 .00			
Change in probability for AQoL utility score <0.37 at each level of the Charlson Score							
	Coeff	95 % CI	*P* Value		Coeff	95 % CI	*P* Value
Charlson Score 2–3*AQOL	0.53	-0.11 to 0.12	0.106	Charlson Score 2–3*AQOL	0.11	0.04 to 0.19	0.003
Charlson Score 4–5*AQOL	0.19	1.00 to 0.28	<0.001	Charlson Score 4–5*AQOL	0.19	0.10 to 0.28	<0.001
Charlson Score (≥6)*AQOL	0.11	-0.12 to 0.22	0.079	Charlson Score ≥ 6*AQOL	0.12	0.01 to 0.23	0.040
Sensitivity	24.85 %	False Negative	75.15 %	Sensitivity	69.7 %	False Negative	30.3 %
Specificity	92.12 %	False Positive	7.88 %	Specificity	52.2 %	False Positive	47.8 %
Positive Predictive value	60.30 %	False Positive	39.70 %	Positive Predictive value	63.3 %	False Positive	36.8 %
Negative Predictive value	71.8 %	False Negative	28.20 %	Negative Predictive value	59.4 %	False Negative	40.6 %
Emergency Inpatient admissions within 1 year				Inpatient admission within 3 years			
Predictor	OR	95 % CI	*P* Value	Predictor	OR	95 % CI	*P* Value
AQOL utility score <0.37	1.25	0.82 to 1.91	0.299	AQOL utility score <0.37	1.67	1.21 to 2. 30	0.002
Charlson Score (2–3)	1.57	1.10 to 2.24	0.012	Charlson Score (2–3)	1.99	1.51 to 2.63	<0.001
Charlson Score (4–5)	2.79	1.87 to 4.18	<0.001	Charlson Score (4–5)	2.62	1.85 to 3.73	<0.001
Charlson Score (≥6)	6.30	4.11 to 9.66	<0.001	Charlson Score (≥6)	8.76	5.59 to 13.73	<0.001
Correct classification: 77.5 %; Hosmer and Lemeshow χ^2^ (8) = 5.71, *p* = 0.456				Correct classification: 68.2 %; Hosmer and Lemeshow χ^2^ (8) = 4.95, *p* = 0 .666			
Change in probability for AQoL utility score <0.37 at each level of the Charlson Score							
	Coeff	95 % CI	*P* Value		Coeff	95 % CI	*P* Value
Charlson Score 2–3*AQOL	0.27	-0.13 to 0.08	0.316	Charlson Score 2–3*AQOL	0.11	0.04 to 0.18	0.003
Charlson Score 4–5*AQOL	0.16	0.08 to 0.25	<0.001	Charlson Score 4–5*AQOL	0.19	0.96 to 0.29	<0.001
Charlson Score (≥6)*AQOL	0.08	-0.03 to 0.19	0.147	Charlson Score ≥ 6*AQOL	0.11	-0.01 to 0.23	0.066
Sensitivity	18.2 %	False Negative	81.8 %	Sensitivity	44.3 %	False Negative	55.7 %
Specificity	96.8 %	False Positive	3.2 %	Specificity	86.2 %	False Positive	13.8 %
Positive Predictive value	65.0 %	False Positive	35.0 %	Positive Predictive value	70.1 %	False Positive	29.9 %
Negative Predictive value	78.5 %	False Negative	21.5 %	Negative Predictive value	67.9 %	False Negative f	32.1 %
				Interaction between HRQoL measured by AQoL and Charlson Score not significant, adjusted by age Not significant			

* = Interaction between Charlson Score and AQoL score in the multivariate model

At one year, the Charlson score was predictive of both ED attendances and inpatient admissions, while AQoL utility score < 0.37 was not predictive after adjusting for interactions with Charlson score (Table 4). Tests of marginal effects demonstrated that in both the model to predict ED attendance and to predict inpatient admission, an AQoL score < 0.37 was a significant predictor for those with Charlson scores of 4–5 but not for those with Charlson Scores < 4 or >5. In contrast, an AQoL utility score < 0.37 was predictive of both ED presentation (Yes/No) and inpatients admissions (Yes/No) over three years after adjusting for differences in age and any interaction between AQoL score and Charlson comorbidity score (Table 5).

## Discussion

This study found that both HRQoL and number of comorbidities (measured by the Charlson Score) were predictive of subsequent emergency department attendance or inpatient admission over three years of follow-up. Patient reported HRQoL was partially related to burden of comorbidities with lower HRQoL reported by those with more comorbidities. At one year, number of comorbidities was a better predictor of emergency department re-attendance and hospital readmission than HRQoL. It is noteworthy that over longer periods of follow-up, patients’ self-reported HRQoL became an independent predictor of the need for acute care services with statistically significant increases in odds of emergency department re-attendance or readmission across all levels of comorbidity burden for those with AQol utility scores < 0.37. The multivariate models had high levels of specificity for patients who would have a hospital re-attendance at one-year (92 % specific) and three-years (97 % specific). The positive predictive values were 60–65 % and false negative rates of 20–28 % meaning that not all patients who would subsequently have an acute care attendance would be identified by these models.

These findings confirm previous studies using disease-specific measures of quality of life that have found an associated between quality of life and acute care re-attendances. For patients with COPD and CHF decrements in the physical functioning dimension of HRQoL has been found be associated with a two to five fold increase in the odds of hospital readmission [9–11]. The association between poor self-reported quality of life and adverse health outcomes may be related to HRQoL being a proxy measure for disease severity, functional decline and burden of comorbidities [34]. Although generic measures of HRQoL for older adults (mean age 81 years) has also been found to predict subsequent hospitalisation independent of disease severity [29–34]. HRQoL measures may also capture underlying psychological constructs such as overall satisfaction with personal circumstances, levels of social support, personal relationships and living environment, all factors which are acknowledged to be predictive of increased ED attendances in frail older adults [31, 32].

Although there are some limitations to calculating comorbidities based on administrative data as there is the possibility of under-reporting of comorbidities during the hospital stay, the Charlson scale is established as having reasonable predictive value for healthcare costs and patient mortality [35]. The association between comorbidity burden, health-related quality of life and acute health care utilisation found in this study confirms previous reports [36]. For COPD patients, comorbidity burden is known to determine both elevated utilisation and acute health care costs with those in the highest quartile for comorbidities consuming 63 % of the acute costs for this condition [37, 38]. For patients with diabetes those with three or more diabetes-related systemic complications had two-fold higher acute care costs compared to those without complications [39].

The main limitations of this study was that it used a sample of patients enrolled in the disease management programs who were willing to give verbal consent to complete the AQoL questionnaire, therefore there may be some bias in the selection of patients included in this analysis. Administrative data as used to obtain acute care utilisation data from the health service that provided the chronic disease management program in which patients were enrolled. It is possible that some patients may have been admitted elsewhere for acute care management during the three years of follow-up.

This study confirms previous research that across a wide range of conditions, adults with chronic disease report lower health-related quality of life than aged matched that population based samples [40]. The magnitude of decrement in HRQoL varied across the different medical conditions, with the highest HRQoL reported by younger adults attending asthma, ischaemic heart disease program and the lowest quality of life reported by those requiring aged care services and those being managed for complicated peripheral vascular disease. These differences reflect the impact of both the primary health condition and the impact of comorbidities, the greatest impacts being demonstrated in the illness and independent living dimensions of the AQoL. Consistent with previous studies, these findings demonstrate that as the population ages and the prevalence of chronic disease increases that the prevalence of individuals reporting lower HRQoL is also likely to increase [40]. Policy makers can therefore anticipate increased demand for both medical management of chronic disease but also importantly increased demand for allied health and supportive care services to assist individuals to adjust to and manage the impact these conditions have on their personal well-being, functional capacity and personal independence [12, 13, 33, 39].

## Conclusion

Measures of HRQoL and burden of comorbidities measure separate risk factors for hospital readmission that also interact with each other. These findings indicate that clinicians evaluating patients’ need for ambulatory chronic disease management programs should take into consideration both their comorbidity burden and the patients’ perception of the impact of their condition(s) on their HRQoL. To maximise their benefits ambulatory disease management programs should initially focus on optimisation of medical management of chronic disease and associated comorbidities, but subsequently the focus should switch to strategies that enhance health independence and raise HRQoL over the longer term.

## Abbreviations

AQoL: Assessment of quality of life
CHF: Chronic heart failure
COPD: Chronic obstructive pulmonary disease
ED: Emergency department
HRQoL: Health-related quality of life
IHD: Ischaemic heart disease
MID: Minimum important difference
NA-HARP: Northern alliance hospital admission risk program
95 %CI: Ninety-five percent confidence interval
OR: Odds ratio
PVD: Peripheral vascular disease
SD: Standard deviation

## References

[1] AIHW (2006). Chronic diseases and associated risk factors in Australia.

[2] Glover J, Page A, Ambrose S, Hetzel D. Atlas of avoidable hospitalisations in Australia: ambulatory care-sensitive conditions. Cat. no. HSE 49. Canberra: AIHW. 2007. Viewed 14 May 2015 <http://www.aihw.gov.au/publication-detail/?id=6442467966>.

[3] Tian Y, Dixon A, Gao H (2012). Data Briefing - Emergency hospital admissions for ambulatory care sensitive conditions: identifying the potential for reductions.

[4] Victorian Department of Human Services. The Victorian Ambulatory Care Sensitive Conditions Study 2001–02. Published by Public Health, Rural and Regional Health and Aged Care Services Division, Victorian Government Department of Human Services Melbourne Victoria July 2004 http://docs.health.vic.gov.au/docs/doc/A595DDEB69465A78CA25787800832646/$FILE/acsc_finalreport.pdf.

[5] Howell S, Coory M, Martin J, Duckett S (2009). Using routine inpatient data to identify patients at risk of hospital readmission. BMC Health Serv Res.

[6] Grunier A, Dhallia IA, van Walraven C, Fischer HD, Camacho X, Rochon PA (2011). Unplanned readmissions after hospital discharge among patients identified as being at high risk for readmission using a validated predictive algorithm. Open Med.

[7] Billings J, Dixon J, Mijanovich T, Wennberg D. Case finding for patients at risk of readmission to hospital: development of an alogithm to identify high risk patients. BMJ. 2006. doi:10.1136/bmj.38870.657917.AE. 10.1136/bmj.38870.657917.AE PMC1539047 16815882

[8] Soler JJ, Sanchez L, Roman P, Martinez MA, Perpina M (2004). Risk factors of emergency care and admissions in COPD patients with high consumption of health respources. Respir Med.

[9] Sprenkle MD, Niewoehner DE, Nelson DB, Kristin L, Nichol KL (2004). The veterans short form 36 questionnaire is predictive of mortality and health-care utilization in a population of veterans with a self-reported diagnosis of asthma or COPD. Chest.

[10] Fan VS, Curtis JR, Tu SP, McDonell MB, Fihn SD, Investigators ACQIP (2002). Using quality of life to predict hospitalization and mortality in patients with obstructive lung diseases. Chest.

[11] Rodriguez-Artalejo F, Guallar-Castillion P, Rodriguez-Pascual C, Montoto C, Ortega Montes A, Nieto Garcia A (2005). Health-related quality of life as a predictor of hospital readmission an death among patients with heart failure. Arch Intern Med.

[12] Bourbeau J, Julien M, Maltais F, Rouleau M, Beaupré A, Bégin R (2003). Reduction of hospital utilization in patients with chronic obstructive pulmonary disease: a disease-specific self-management intervention. Arch Intern Med.

[13] Annema C, Luttik M-L, Jaarsma T (2009). Reasons for readmission in heart failure: perspectives of patients, caregivers, cardiologists, and heart failure nurses. Heart Lung.

[14] Krumholz HM, Chen Y-T, Wang Y, Vaccarino V, Radford MJ, Horwitz RI (2000). Predictors of readmission among elderly survivors of admission with heart failure. Am Heart J.

[15] Harvey P, Storer M, Berlowitz DJ, Jackson B, Hutchinson AF, Lim WK (2014). Feasibility and impact of a post–discharge geriatric evaluation and management service for patients from residential care: the Residential Care Intervention Program in the Elderly (RECIPE). BMC Geriatr.

[16] Berlowitz DJ, Graco M (2010). The development of a streamlined, coordinated and sustainable evaluation methodology for a diverse chronic disease management program. Aust Health Rev.

[17] Hutchinson AF, Rasekaba TM, Graco M, Berlowitz DJ, Hawthorne G, Lim WK (2013). Relationship between health-related quality of life, and acute care re-admissions and survival in older adults with chronic illness. Health Qual Life Outcomes.

[18] Hawthorne G, Richardson J, Osborne R (1999). The Assessment of Quality of Life (AQoL) instrument: a psychometric measure of health-related quality of life. Qual Life Res.

[19] Osborne RH, Hawthorne G, Lew EA, Gray LC (2003). Quality of Life assessment in the community-dwelling elderly: validation of the Assessment of Quality of Life (AQoL) instrument and comparison with the SF-36. J Clin Epidemiol.

[20] Hawthorne G, Osborne R (2005). Population norms and meaningful differences for the Assessment of Quality of Life (AQoL) measure. Aust N Z J Public Health.

[21] Hawthorne G, Korn S, Richardson JR. Population norms for the AQoL derived from the 2007 Australian National Survey of Mental Health and Wellbeing. Aust N Z J Public Health. In press; Accepted 18 October. 10.1111/1753-6405.12004 23379800

[22] WHO (1993). The ICD-10 Classification of Mental and Behavioural Disorders Diagnostic Criteria for Research.

[23] Quan H, Sundararajan V, Halfon P, Fong A, Burnand B, Luthi JC (2005). Coding algorithms for defining comorbidities in ICD-9-CM and ICD-10 administrative data. Med Care.

[24] Charlson ME, Pompei P, Ales KL, MacKenzie CR (1987). A new method of classifying prognostic comorbidity in longitudinal studies: development and validation. J Chronic Dis.

[25] Welch BL (1947). The generalization of “Student’s” problem when several different population variances are involved”. Biometrika.

[26] UCLA: Statistical Consulting Group. Stata FAQ. How can I use the margins command to understand multiple interactions in logistic regression? (Stata 11). from http://www.ats.ucla.edu/Stat/stata/faq/margins_mlogcatcon.htm/ (accessed December 19th, 2014.

[27] Hosmer DW, Lemeshow S (2000). Applied Logistic Regression.

[28] Stata Statistisical Software, StatCorp LB. Texas, USA. http://www.stata.com/.

[29] Banham D, Hawthorne G, Goldney R, Ratcliffe J (2014). Health-related qulaity of life (HRQoL) changes in South Australia: comparsion of burden of disease morbidity and survey-based health itility estimates. Health Qual Life Ouctomes.

[30] Bilotta C, Bowling A, Nicolini P, Case A, Pina G, Rossi SV (2011). Older people’s Quality of Life (OPQOL) scores and adverse health outcomes at a one-year follow-ip A prospectove cohort study on older outpatients in the community in Italy. Health Qual Life Outcomes.

[31] Cavrini, G., Broccoli S, Puccini A, Zoli B. EQ-5D as a predictor of mortality and hospitalization in elderly people. Qual Life Res. 2012;21(2):269-80. doi:10.1007/s11136-011-9937-0. Epub 2011 Jun 9. 10.1007/s11136-011-9937-0 21656336

[32] Shim EU, Ma SH, Hong SH, Lee YS, Paik WY, Seo DS (2011). Correlation between frailty level and adverse health-related outcomes of community-dwelling elderly, one year retrospective study. Korean J Fam Med.

[33] Bilotta C, Bowling A, Case A, Nicolini P, Mauri S, Castelli M (2010). Dimensions and correlates of quality of life according to frailty status: a cross-sectional study on community-dwelling older adults referred to an outpatient geriatric service in Italy. Health Qual Life Outcomes.

[34] Bowling A, Grundy E (2009). Differentials in mortality up to 20 years after baseline interview among older people in East London and Essex. Age Ageing.

[35] Perkins AJ, Kroenke K, Unutzer J, Katon W, Williams JW, Hope C (2004). Common comorbidity scales were similar in their ability to predict health care costs and mortality. J Clin Epidemiol.

[36] Agborsangaya CB, Lau D, Lahtinen M, Cooke T, Johnson JA (2012). Multimorbidity prevalence and patterns across socioeconomic determinats: a cross-sectional survey. BMC Public Health.

[37] Simon-Tuval T, Scharf SM, Maimon N, Bernhard-Scharf BJ, Reuven H, Tarasiuk A. Determinants of elevated healthcare utilization in patients with COPD. Respir Res. 2011;12:7. http://respiratory-research.com/content/12/1/7. 10.1186/1465-9921-12-7 PMC3032684 21232087

[38] Ng T-P, Niti M, Tan W-T, Cao Z, Ong K-C, Eng P (2007). Depressive symptoms and chronic obstructive pulmonary disease. Effect on mortality, hospital readmission, symptom burden, functional status, and quality of life. Arch Intern Med.

[39] Simon GE, Katon WJ, Lin EHB, Ludman E, von Korff M, Ciechanowski P, et al. Diabetes complications and depression as predictors of healthcare costs. General Hospital Psychiatry, 2005, 344–351. doi:10.1016j.genhosppsych.2005.04.008. 10.1016/j.genhosppsych.2005.04.008 16168795

[40] Walker AE (2007). Multiple chronic diseases and quality of life: patterns emerging from a large national sample, Australia. Chronic Illn.

